# Achievable rate analysis of orbital angular momentum multiplexing and demultiplexing using E-band metasurfaces

**DOI:** 10.1038/s41598-026-40149-7

**Published:** 2026-02-19

**Authors:** Hyeongju Chung, Beomjoon Kim, Young-Seok Lee, Hongeun Choi, Bang Chul Jung, Eunmi Choi, Jongwon Lee

**Affiliations:** 1 Department of Electrical Engineering, Ulsan National Institute of Science and Technology (UNIST), Ulsan, 44919 Republic of Korea; 2 Department of Artificial Intelligence Convergence Network, Ajou University, Suwon, 16499 Republic of Korea; 3 Department of Electrical and Computer Engineering, Ajou University, Suwon, 16499 Republic of Korea

**Keywords:** Metasurface, Orbital angular momentum, E-band, Engineering, Optics and photonics, Physics

## Abstract

To meet the growing demand for high-capacity wireless communications, orbital angular momentum (OAM) multiplexing has garnered significant attention due to the orthogonality between OAM modes, which enables enhanced channel capacity. In this work, we propose and experimentally demonstrate a metasurface-based OAM mode-division multiplexing (OAM-MDM) system operating in the E-band. The system employs Fabry–Perot cavity meta-atoms that offer high transmission efficiency and precise phase control, enabling metasurfaces capable of multiplexing and demultiplexing two distinct OAM modes. We establish an electromagnetic-based effective channel model that characterizes the magnitude and phase variations between transmitted and received OAM modes from the radiated electric field. Specifically, the proposed effective wireless channel model captures not only the desired mode-to-mode transmission but also the inter-mode interference and represents these effects in a mathematically tractable form suitable for communication-theoretic analysis. Furthermore, the system performance is comprehensively evaluated by comparing the achievable rates derived from both simulations and experimental measurements under varying input power levels. Experimental results demonstrate that an achievable rate of up to 41.8 bits/s/Hz is attained at an input power of 4.9 dBm. This metasurface-based OAM-MDM system presents a promising approach for future high-capacity free-space communication.

**Supplementary Information:**

The online version contains supplementary material available at 10.1038/s41598-026-40149-7.

## Introduction

With the explosive growth of data demand driven by emerging technologies such as augmented reality, artificial intelligence and the Internet of Things (IoT), conventional multiplexing strategies—based on amplitude^1,2^, frequency^3,4^, and polarization^5^—are approaching their physical limits in terms of spectral efficiency and channel capacity. To address this growing demand, orbital angular momentum (OAM) has gained increasing attention as a new degree of freedom for multiplexing in wireless communications^6–8^. OAM, which can be described as a subset of Laguerre–Gaussian beams^9^, is characterized by a doughnut-shaped intensity profile and a helical phase wavefront, mathematically represented by $\:exp\left(il\varphi\:\right)$, where $\:l$ is an integer number denoting the OAM mode index and *ϕ* is the azimuthal angle. A key advantage of OAM multiplexing lies in the mutual orthogonality between modes, theoretically enabling unlimited channel capacity under ideal conditions.

Two major approaches have been widely adopted for experimental implementation of OAM multiplexing: uniform circular array (UCA) antennas^10,11^ and metasurfaces^12–14^. UCA-based OAM multiplexing has demonstrated impressive performance in sub-terahertz bands, achieving wireless transmission rates of up to 1.58 Tbps for future 6G backhaul and fronthaul networks^15^. However, UCAs have limitations in integrating additional functionalities such as multi-mode beam generation, beam steering, and focusing within a compact platform. To overcome these constraints, metasurface-based approaches have emerged as a promising alternative. Metasurfaces, composed of subwavelength-scale meta-atoms, allow for local manipulation of light’s amplitude, phase, and polarization, enabling a wide range of applications such as metalenses^16,17^, waveplates^18^, optical tweezers^19^, and holography^20^ and reconfigurable intelligent surfaces (RIS)^21–23^. Besides these two major approches, alternative implementations of OAM multiplexing using non-uniform traveling-wave current sources^24,25^ have also been reported. In the context of metasurface-based OAM multiplexing, our group has previously demonstrated an E-band OAM multiplexing and demultiplexing system using metasurfaces^26^. More recently, OAM index modulation schemes have also been experimentally demonstrated using metasurface-based OAM multiplexing^27^.

**Fig. 1:**
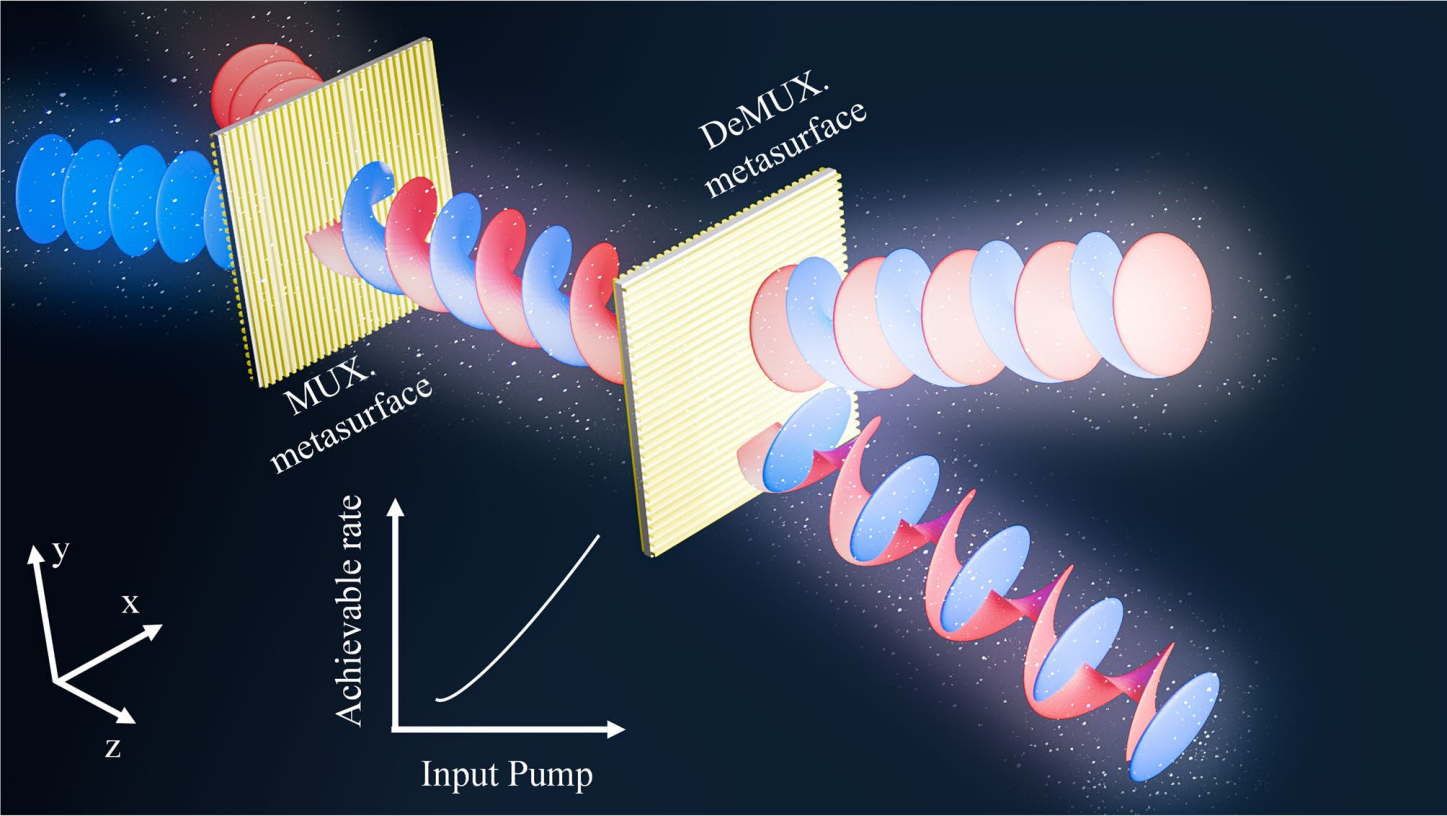
Schematic illustration of the OAM mode-division multiplexing communication system. The red and blue colors represent two distinct data-carrying beams. After passing through multiplexing (MUX) and demultiplexing (DeMUX) metasurfaces, the data encoded in the two incident Gaussian beams are retrieved as off-axis Gaussian beams. The achievable communication rate of the system can be enhanced by increasing the input pump power.

In this work, we propose and experimentally realize a metasurface-based OAM-mode division multiplexing (OAM-MDM) system operating in the E-band as illustrated in Fig. 1. Unlike our previous E-band metasurface-based OAM studies that focused on device-level feasibility using a single incident source, the present work targets a system-level and communication-oriented investigation of OAM multiplexing and demultiplexing. A Fabry–Perot-like cavity meta-atom is designed to achieve improved transmission efficiency and enhanced phase tunability compared to our previous design^26^. The transmitter-side metasurface integrates OAM phase profiles with beam-steering functionality to generate multiplexed OAM beams from two simultaneously incident sources. On the receiver side, the metasurface further incorporates a metalens phase profile to focus the multiplexed OAM beams and spatially separate them for effective demultiplexing, thereby enabling true multi-source OAM-MDM operation with overlapping beams. Beyond device demonstration, we establish an effective wireless channel model that characterizes the interaction between transmitted and received OAM modes in the proposed OAM-MDM system based on the radiated electric field. This model quantitatively describes the transmission characteristics, including magnitude and phase variations between the desired modes, while also accounting for inter-mode interference. It provides a mathematically tractable framework for analyzing OAM-MDM systems from a communication theory perspective. Based on this effective channel model, we evaluate the information-theoretic performance by comparing the achievable rates obtained from simulations and experimental measurements under different input power levels. The results confirm the practical feasibility and effectiveness of metasurface-based OAM multiplexing for future high-capacity wireless communication systems.

## Results and discussion

**Fig. 2:**
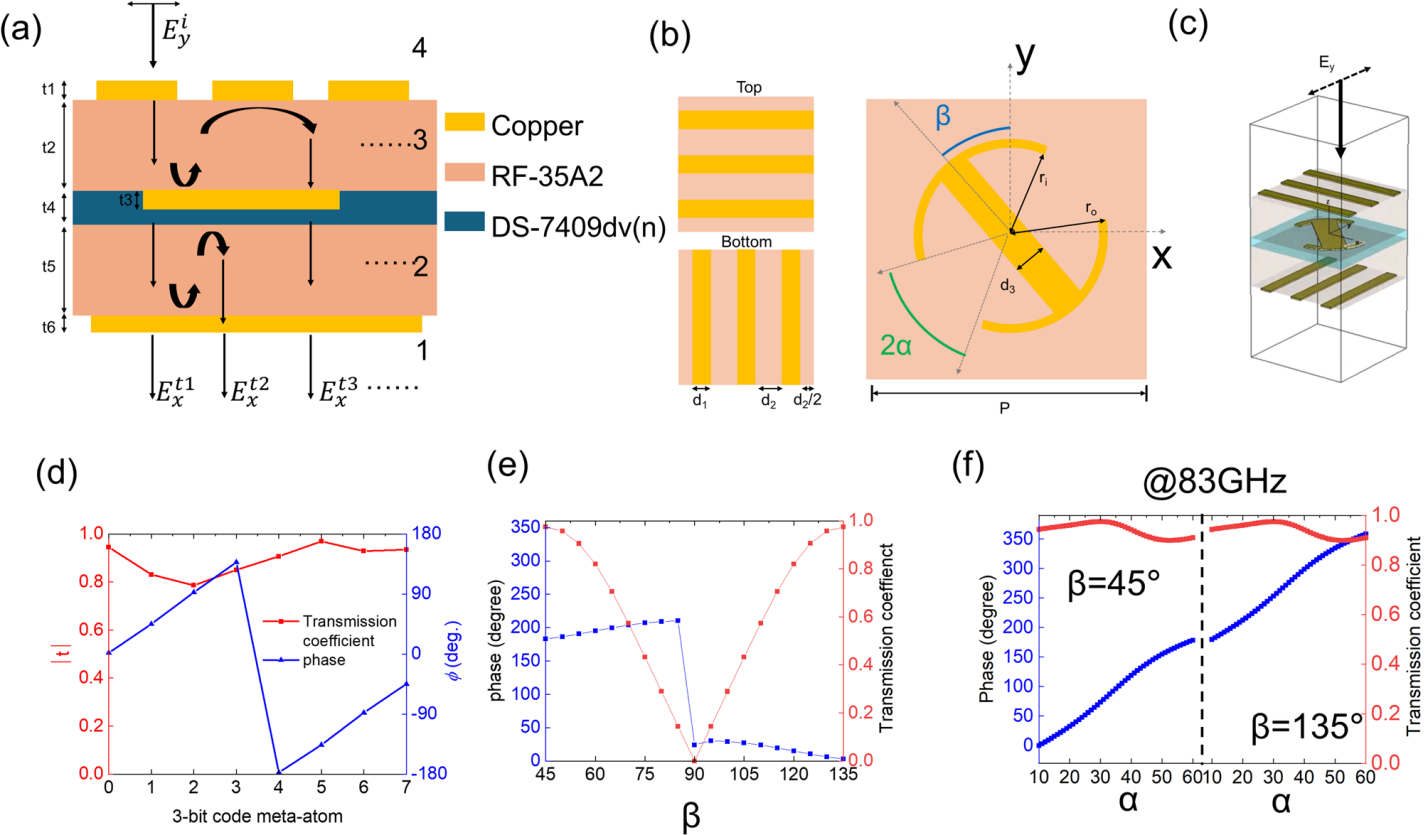
(**a**) Cross-sectional view of the Fabry-Perot like cavity meta-atom. It is composed of three copper layers (thickness: 0.018 mm, t_1_,t_3_, t_6_), two RF-35A2 substrates (thickness: 0.510 mm, t_2_,t_4_) and one bonding material (Ds-7409dv(n) 3313, thickness: 0.108 mm, t_3_). (**b**) Top-view shapes of each copper layer in the meta-atom. In the middle copper layer, 2α is the angle between wings of the I-shape, and β is the rotation angle between the y-axis and the I-shape. The specific dimensions are d_1_ = 0.14, d_2_ = 0.33, d_3_ = 0.3, r_i_=0.45 and r_o_=0.53 (unit: mm). (**c**) Schematic of the meta-atom design with independently controlled amplitude and phase. (**d**) Transmission coefficient$\:{(T}_{xy})$ and phase reseponse of a previously designed meta-atom. (**e**,**f**) Transmission coefficient $\:{(T}_{xy})$ and phase variation as functions of angles α and β, respectively.

We design a meta-atom unit structure based on a Fabry-Perot-like cavity^28,29^ that enables precise phase control while maintaining consistently high transmission efficiency, in contrast to previously designed meta-atoms based on the transfer matrix method^26^. As shown in Fig. 2(a), the meta atom structure consists of three copper layers, two dielectric substrates (RF-35A2, with a dielectric constant of 3.5 and a loss tangent of 0.0018), and one bonding film (DS-7409DV(n), with a dielectric constant of 3.3 and a loss tangent of 0.003). The specific geometries of each copper layer are illustrated in Fig. 2(b). The top and bottom copper layers are composed of three x- and y-directional gratings with identical widths, while the middle copper layer is composed of I-shape. Here, 2α denotes the angle between the wings of I-shape, and β represents the rotation angle between the I-shape and the y-axis. In this structure, the top and bottom grating layers primarily transmit the *E*_*y*_ and *E*_*x*_ field components, respectively. The overall three-dimensional configuration of the proposed meta-atom, including the stacked copper and dielectric layers, is shown in Fig. 2(c). By optimizing the geometry of the middle I-shaped copper layer, the structure exhibits high-efficiency cross-polarization conversion (*T*_*xy*_), resulting from multiple reflections and transmissions within the Fabry-Perot-like cavity, as illustrated in Fig. 2(a) and described by the following equation:

1 $$\:{E}_{x}^{t}={(T}_{xy})\cdot \:{E}_{y}^{i}=({t}_{yy}^{34}{t}_{xy}^{23}{t}_{xx}^{12}{e}^{i\phi\:1}+{t}_{yy}^{34}{t}_{yy}^{23}{r}_{yy}^{12}{r}_{xy}^{32}{t}_{xx}^{12}{e}^{i\phi\:2}+{t}_{yy}^{34}{r}_{xy}^{23}{r}_{xx}^{43}{t}_{xx}^{23}{t}_{xx}^{12}{e}^{i\phi\:3}+\cdots\:){E}_{y}^{i}$$

where, $\:{E}_{x}^{t}$, $\:{E}_{y}^{i}$ represent the transmitted electric field in the x-direction and the incident electric field in the y-direction, respectively. The superscript *ij* denotes the transmission(*t*) or reflection(*r*) from layer *j* to layer *i*, while the subscript *xy* refers to polarization conversion from y- to x-direction. Compared to the simulated transmission coefficient and phase response of eight previously designed meta-atoms as shown in Fig. 2(d)^26^, the proposed meta-atom exhibits improved transmission efficiency and enhanced phase controllability. Notably, only two parameters of the middle copper layer are required to independently control both amplitude and phase. The transmission coefficient of $\:{T}_{xy}$ can be tuned by varying the rotation angle *β* from 45° to 135°, as shown in Fig. 2(e). While *β* has little effect on the phase of $\:{T}_{xy}$, a 180° phase flip occurs when *β* crosses 90°, due to the anti-symmetric mode of the I-shape antenna structure^14,30^. As a result, there is an approximate π-phase difference between the ranges of 45°–90° and 90°–135°. The maximum transmission coefficient is achieved at *β* = 45° or 135°. The phase of $\:{t}_{xy}$ can be further tuned by adjusting the opening angle *α* from 10° to 65° as shown in Fig. 2(f), while fixing *β* = 45° to maintain high transmissive efficiency. This approach enables a phase range of 0° to 180°, and remaining 180° to 360° phase range can be achieved by simply switching *β* from 45° to 135°, leveraging the anti-symmetric mode characteristic. Consequently, a full 360° phase coverage can be achieved while maintaining a transmission coefficient above 0.9. For the high-efficiency design of the metasurface, a total of 72 meta-atoms were selected with uniform phase intervals of 5°, with *β* fixed at either 45° or 135°.

**Fig. 3:**
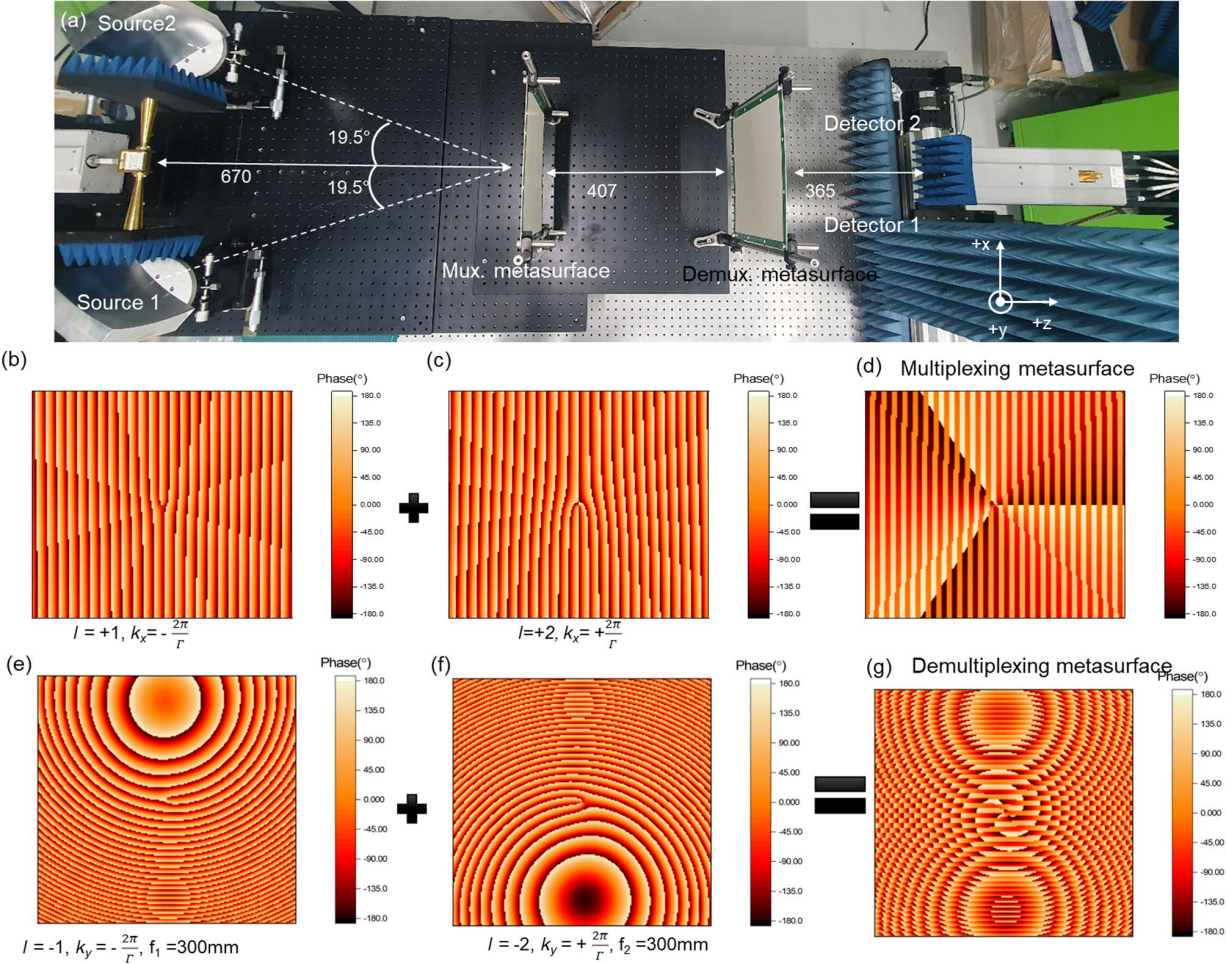
(**a**) Experiment set-up of the OAM-MDM communication system. (**b**) Phase pattern for OAM mode *l* = 1 combined with a one-dimensional (1D) gradient pattern in the –x direction. (**c**) Phase pattern for OAM mode *l* = 2 combined with a 1D gradient pattern in the + x direction. (**d**) Multiplexing phase pattern formed by combining (**b**) and (**c**). (**e**) Phase pattern for OAM mode *l*=-1 combined with a 1D gradient in the –y direction and a lens phase pattern with focal length f_1_=300 mm. (**f**) Phase pattern for OAM mode *l*=-2 combined with a 1D gradient in the + y direction and a lens phase pattern with focal length f_2_=300 mm. (**g**) Demultiplexing phase pattern formed by combining (**e**) and (**f**).

For the multiplexing and demultiplexing of OAM modes *l* = + 1, + 2, the transmission functions of the corresponding metasurfaces are designed as follows:

2 $$\:{M}_{P}\left(x,y\right)={\sum\:}_{P=\mathrm{1,2}}{A}_{P}\left(x,y\right){exp}j\left({l}_{P}{\mathrm{tan}}^{-1}\left(\frac{y}{x}\right)+{k}_{xP}x\right)$$

3 $$\:{M}_{Q}(x,y,\phi\:)={\sum\:}_{Q=\mathrm{1,2}}{A}_{Q}(x,y){exp}j\left({l}_{Q}{\mathrm{tan}}^{-1}\left(\frac{y}{x}\right)+{k}_{yQ}x+{\phi\:}_{Q}\right)$$

where x and y are the spatial coordinate of the metasurface. $\:{A}_{P\:or\:Q}\left(x,y\right)$ represents spatial transmission coefficient at position $\:(x,y)$ for the P^th^ or Q^th^ beam. Here, the indices P and Q both indicate the incident beam source. For example, *P* = 1(or Q = 1) corresponds to source 1 and *P* = 2 (or Q = 2) corresponds to source 2, as shown in Fig. 3(a). In this work, $\:{A}_{P\:or\:Q}\left(x,y\right)$ is assumed to be constant amplitude, as the meta-atom unit cell exhibits uniform transmission coefficient. The parameters $\:{l}_{P\:or\:Q}$,$\:\:{k}_{xP\:or\:yQ}$, $\:{\phi\:}_{Q}$ denote the OAM topological charge, the x or y-directional wavevector of the P^th^ or Q^th^ beam, and the lens phase of the Q^th^ beam, respectively. The experimental set-up for the OAM communication system is illustrated in Fig. 3(a). An E-band signal is split into two beams using a power divider, which is connected to horn antennas. The resulting Gaussian beams are reflected by parabolic mirrors and directed toward the multiplexing metasurface at incident angles of ± 19.5° relative to the z-axis. To generate the two OAM beams with *l* = + 1 and + 2, the phase pattern shown in Fig. 3(d) is applied for the multiplexing metasurface. This pattern is constructed by superimposing the phase profiles of Fig. 3(b) (OAM with *l*_1_ = + 1 and + x directional transverse wavevector of k_x1_=2π/Γ) and Fig. 3(c) (OAM with *l*_2_ = + 2 and -x directional transverse wavevector of k_x2_=-2π/Γ, where Γ = 1.35mm$\:\times\:$8 = 10.8 mm is the period of the 1D phase gradient). The generated OAM beams are then directed to the demultiplexing metasurface, which separates them into ± y directions at ± 19.5° relative to the z-axis. To recover the generated OAM beams, the phase pattern shown in Fig. 3(g) is applied for the demultplexing metasurface. This pattern is formed by superimposing the phase profiles of Fig. 3(e) (OAM with *l*_1_=-1, -y directional transverse wavevector of k_y1_=-2π/Γ, and lens phase with focal length of f_1_=300 mm) and Fig. 3(f) (OAM with *l*_2_=-2, +y directional transverse wavevector k_y2_ = + 2π/(1.35$\:\times\:$8mm) and lens phase with focal length of f_2_=300 mm). The focal length of the demultiplexing metasurface was chosen as 300 mm based on the practical constraints of the experimental setup and the available propagation distance in the E-band measurement system. Due to cumulative beam divergence, the effective focal plane was experimentally identified at approximately 365 mm.

The generation and reception of metasurface-based OAM beams were simulated using the Angular Spectrum Method (ASM)^31–33^instead of CST full-wave simulations. ASM computes beam propagation by decomposing the complex wave field into an infinite number of plane waves, which are then propagated to a target location. Compared to CST simulation, which demands high memory and long computation times, ASM offers significantly lower memory consumption and faster computation while maintaining sufficient accuracy. A more detailed explanation of ASM is provided in Supporting Information Note 1.

**Fig. 4:**
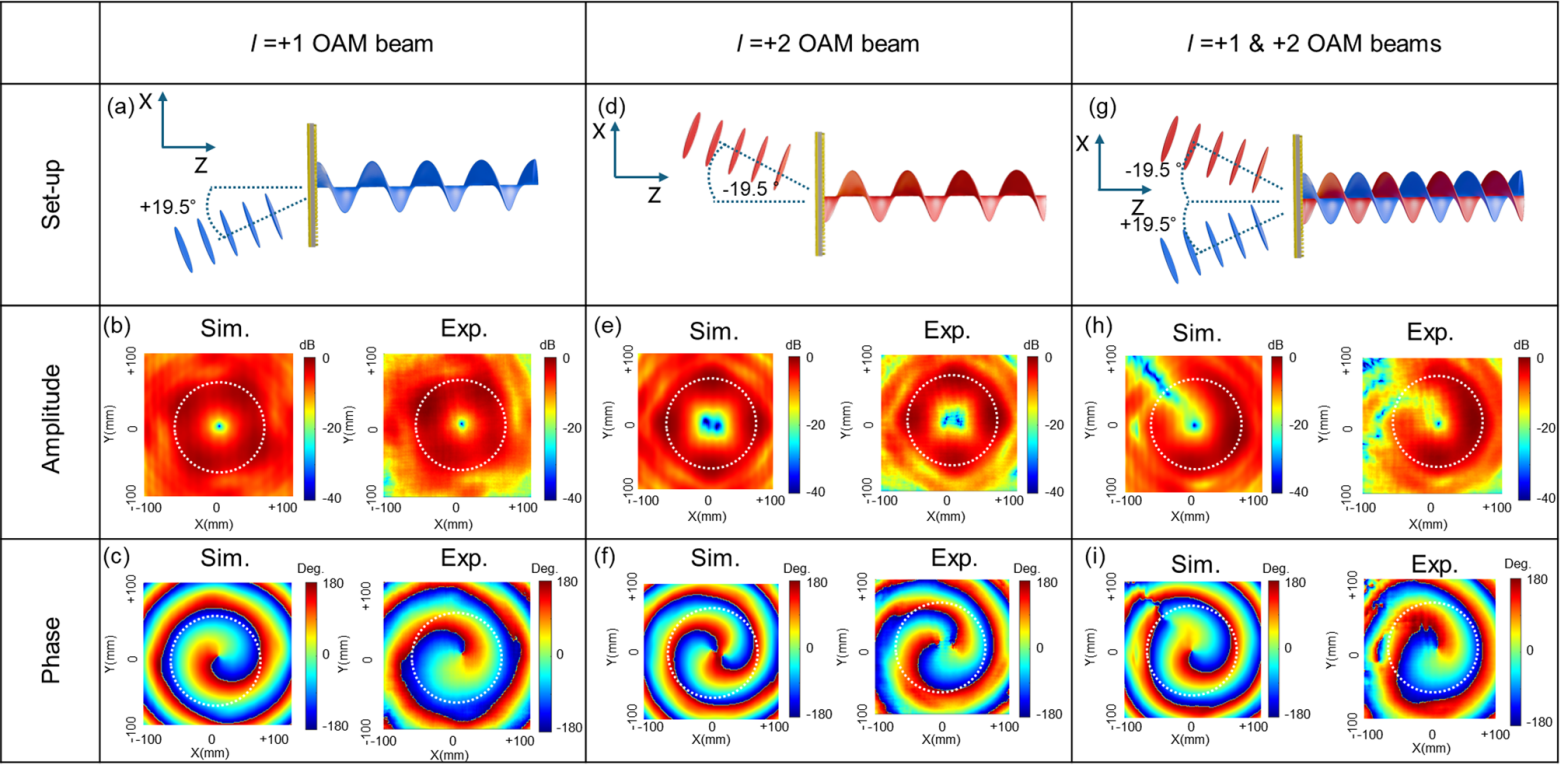
(**a**) Measurement set-up fort he generation of OAM mode *l* = + 1. (**b**,**c**) Simulated and measured amplitude (**b**) and phase (**c**) distributions for OAM mode *l* = + 1. (**d**) Measurement set-up for the generation of OAM mode *l* = + 2. (**e**,**f**) Simulated and measured amplitude (**e**) and phase (**f**) distributions for OAM *l* = + 2. (**g**) Measurement set-up for the simultaneous generation of OAM modes *l* = + 1 and *l* = + 2. (**h**,**i**) Simulated and measured amplitude (**h**) and phase (**i**) distributions for the simultaneous generation of OAM modes *l* = + 1 and *l* = + 2.

The simulation and experimental results of the multiplexing metasurface designed to generate coaxial OAM beams are presented in Fig. 4. To verify the independent generation of OAM modes *l* = + 1 and *l* = + 2, a single Gaussian beam was incident onto the multiplexing metasurface at off-axis angles of ± 19.5°, as shown in the setups of Fig. 4(a) and (d). The resulting amplitude and phase distributions of the generated beams at 83 GHz are shown in Fig. 4(b) and (e) (amplitude) and Fig. 4(c) and (f) (phase) for both simulation and experiment. The observation plane was set to 100 mm × 100 mm and positioned 400 mm away from the metasurface. The generated OAM beams exhibit a uniform doughnut-shaped amplitude profile without significant distortion. In the phase profiles, the *l* = 1 mode exhibits a single 360° counterclockwise helical twist, while the *l* = 2 mode completes two full 360° twists, consistent with theoretical expectations. As illustrated in the schematic in Fig. 4(g), when two Gaussian beams are simultaneously incident at ± 19.5° off-axis angles, interference occurs between the coherent OAM beams^34^. This interference leads to partial intensity cancellation in certain regions, as shown in Fig. 4(h). In the corresponding phase distributions shown in Fig. 4(i), a slight discrepancy between the simulation and experimental results is observed, which is attributed to misalignment of the metasurfaces during the measurement process. In addition, the OAM mode purity was analyzed to quantitatively assess the quality of the generated beams. The circular regions indicated by the white dotted curves in Fig. 4 correspond to the main intensity distributions of the generated fields, and the OAM mode calculation methodology and purity results are provided in Supplementary Note 2 and Figure S2. A detailed sensitivity analysis of the misalignment effect and its impact on the field distributions is provided in the Supporting Information.(see Ssupporting information Note 3 and Figure S3).

**Fig. 5:**
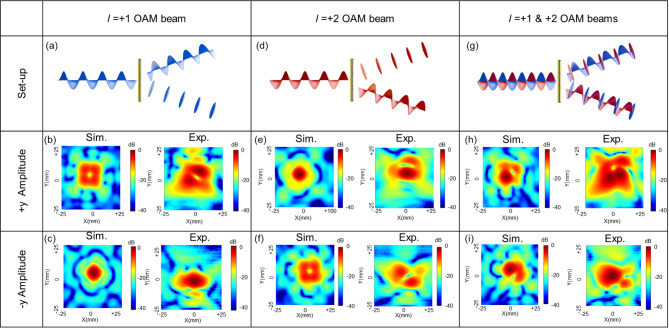
(**a**) Measurement set-up for the demultiplexing of OAM mode *l* = + 1. (**b**,**c**) Simulated and measured amplitude distributions of the demultiplexed OAM mode *l*=-1 (**b**) and the Gaussian beam (**c**). (**d**) Measurement set-up for the demultiplexing of OAM mode *l* = + 2. (**e**,**f**) Simulated and measured amplitude distributions of the Gaussian beam (**e**) and OAM mode *l* = + 1 (**f**). (**g**) Measurement set-up for the demultiplexing of combined OAM modes *l* = + 1 and *l* = + 2. (**h**,**i**) Simulated and measured amplitude distributions of the combined OAM mode *l*=-1 and Gaussian beam (**h**), and the combined Gaussian beam and OAM mode *l* = + 1 (**i**).

To spatially separate the generated OAM beams (*l* = + 1 and + 2) from the multiplexing metasurface, Fig. 5 presents the simulated and measured amplitude distributions at positions where the beams are spatially separated along the ± y-axis by the demultiplexing metasurface. The observation areas were set to 25 mm × 25 mm and located 365 mm away from the demultiplexing metasurface.Using the measurement setups shown in Fig. 5a, d, each of the *l* = + 1 and *l* = + 2 OAM beams is converted into an *l* = − 1 OAM beam and a Gaussian beam in the + y direction (see Fig. 5(b) and 5(e)), and into a Gaussian beam and an *l* = + 1 OAM beam in the –y direction (see Fig. 5c, f). The corresponding amplitude distributions obtained from both simulation and measurements are presented in the figures. Furthermore, when both OAM beams *l* = + 1 and + 2 are incident simultaneously—as illustrated in the schematic in Fig. 5g—interference occurs between the overlapping OAM and Gaussian beams in each ± y-directional detection region, similar to the phenomenon observed in Fig. 4h. The corresponding measured amplitude distributions are shown in Fig. 5h, i, clearly illustrating the interference effects. Overall, a good agreement is observed between the simulation and experimental results.

Regarding metasurface-based OAM-MDM communication system, as illustrated in Fig. 3a, two off-axis Gaussian beams (Source 1 and 2) are directed via a parabolic mirror and sequentially pass through the multiplexing and demultiplexing metasurfaces. At the receiving plane, the resulting transformed OAM and Gaussian beams are spatially separated along the ± y-direction.

These spatial separated beams ensure that the output Gaussian beams, obtained through demultiplexing of the OAM beams, are clearly distinguished according to the incident direction of the input Gaussian beams. Accordingly, by placing detectors at the positions where the Gaussian beam intensity is expected to be the strongest, the incident source can be identified. For example, when only Gaussian Source 1 (or 2) is incident, a signal is observed exclusively at Detector 1 (or 2), whereas simultaneous incidence of both sources results in signal detection at both detectors. In this paper, it is assumed that the inter-beam interference after the demultiplexing metasurface is negligible. Nevertheless, when both sources are incident simultaneously, each detector receives not only the desired Gaussian beam but also interference components induced by the OAM beams originating from the other source. The intra-beam interference appears in the form of OAM mode beams, which exhibit a doughnut-shaped distribution with the phase rotating by an integer multiple of $\:2\pi\:$ depending on the mode index. When equal-gain combining is applied over the detector area, these spatially rotating phase components cancel each other out, thereby eliminating their impact on the received signal.(Figure S2) In this OAM-MDM structure, we aim to define an effective wireless channel that characterizes not only the desired channel from the input source to the output source but also the impact of interference channels. This effective channel can be represented in terms of the electric field measurable at the detector.

The effective channel $\:{H}_{mn}$ represents the channel observed at the $\:m$th detector due to the electric field originating from the $\:n$th source. When only the $\:n$th source is incident, the electric field after the demultiplexing metasurface is defined as $\:{E}_{n}(x,y)$. Accordingly, $\:{H}_{mn}$ can be obtained by applying equal-gain combining of the $\:{E}_{n}(x,y)$ components at the $\:m$th detector, as follows

4 $$\:{H}_{mn}={\int\:}_{y:\left|y-{y}_{m}\right|\le\:R/2}^{}{\int\:}_{x:\left|x-{x}_{m}\right|\le\:R/2}\frac{1}{\sqrt{{R}^{2}}}{E}_{n}\left(x,y\right)dxdy$$

where $\:R$ denotes the side length of the square detector area (set to 3 mm), while $\:\left({x}_{m},{y}_{m}\right)$ represent the coordinates of the beam center at the $\:m$th detector position. To investigate the communication-theoretic meaning of the effective channel, let $\:{s}_{n}$ ​denote the transmitted signal from the $\:n$th source to the receiver, $\:{y}_{m}$ the received signal at the $\:m$th detector, and $\:{w}_{m}$ the thermal noise introduced at the receiver when detecting $\:{y}_{m}$. The received signal can then be expressed as follows

5 $$\:{y}_{m}=\sum\:_{n=1}^{N}{H}_{mn}{s}_{n}+{w}_{m}$$

In vector form, the received signal can be expressed as $\:\mathbf{y}\left(\in\:{\mathbb{C}}^{M}\right)=\mathbf{H}\mathbf{s}+\mathbf{w}\:$where $\:\mathbf{H}\left(\in\:{\mathbb{C}}^{M\times\:N}\right)$ denotes the channel matrix whose $\:\left(m,\:n\right)$th element is given $\:{H}_{mn}$, $\:\mathbf{s}\left(\in\:{\mathbb{C}}^{N}\right)$ denotes the transmit signal vector originating from the sources, and $\:\mathbf{w}\left(\in\:{\mathbb{C}}^{M}\right)$ represents the noise vector at the receiver. This can be confirmed to be identical to the received signal model in conventional multiple-input multiple-output (MIMO) communication systems. One of the most important aspects in information theory is the mathematical modeling of the relationship between inputs and outputs. Based on the defined channel model, the information-theoretic performance of the OAM-MDM system can thus be derived as follows^35^

6 $$\:\mathrm{A}\mathrm{c}\mathrm{h}\mathrm{i}\mathrm{e}\mathrm{v}\mathrm{a}\mathrm{b}\mathrm{l}\mathrm{e}\:\mathrm{R}\mathrm{a}\mathrm{t}\mathrm{e}={\mathrm{log}}_{2}\left\{\mathrm{det}\left({\mathbf{I}}_{N}+\mathbf{H}{\mathbf{H}}^{H}\right)\right\}$$

where, det(⋅) denotes the determinant operator, **I**_*N*_ is the *N × N* identity matrix. In this formulation, the power scaling implied by the field-based channel definition is already reflected in the channel matrix, and no additional normalization is introduced in the performance analysis.

**Fig. 6:**
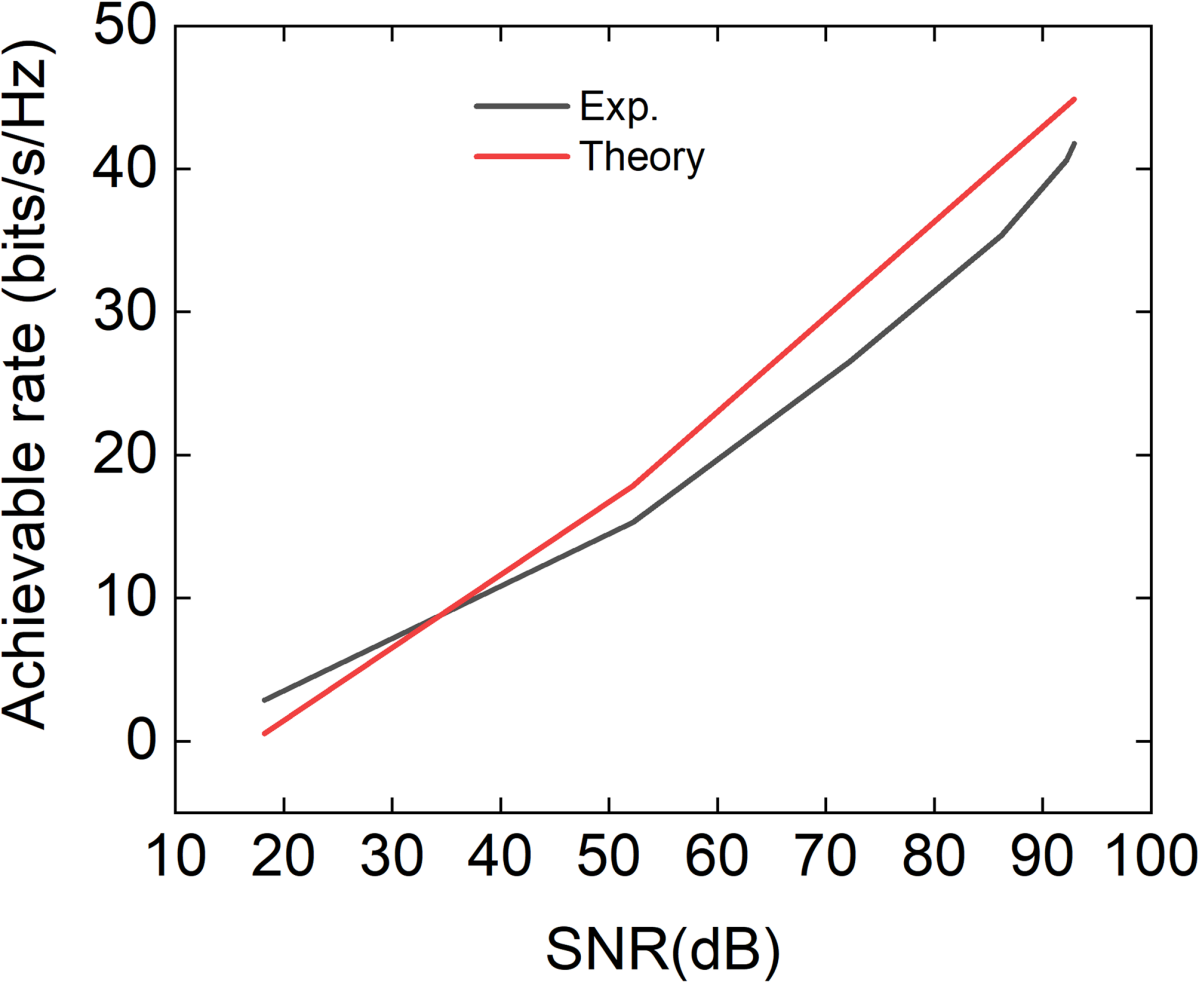
Achievable data rate from simulation and experiment as a function of signal-to-noise ratio (SNR), obtained by varying the input pump power.

To experimentally evaluate the achievable rate, the input power was varied from − 69.8 dBm to 4.9 dBm using a calibrated variable attenuator, and the corresponding achievable rate was extracted as a function of the signal-to-noise ratio (SNR). The comparison between theory and experimental results is shown in Fig. 6. In the experiment, the noise power was directly measured to be − 82.8 dBm and the same noise level was consistently used in the numerical simulations to ensure a fair comparison. As shown in Fig. 6, the achievable-rate curves obtained from theory and experiment exhibit a similar slope with respect to SNR, indicating that the proposed channel model accurately captures the power scaling behavior of the system. Experimentally, a maximum achievable rate of 41.8 bits/s/Hz was obtained at an input power of 4.9 dBm, while the corresponding simulated value is slightly higher. In the low-SNR regime, the experimentally extracted achievable rate is slightly higher than the theoretical prediction. The observation that the measured performance slightly exceeds the theoretical prediction in the low-SNR regime can be attributed to a fundamental difference between the theoretical SNR definition and the practical measurement process. In theoretical analysis, the SNR is defined based on the statistical properties of noise and represents an average quantity. In contrast, experimental measurements rely on instantaneous signal observations over a finite number of samples. When the received signal level approaches the noise floor, as in the low-SNR regime, fluctuations in noise power and measurement uncertainty make accurate estimation of the effective SNR inherently difficult. As a result, the instantaneous measured performance may appear higher than the theoretically predicted average performance in certain cases. This behavior does not indicate a limitation of the theoretical model, but rather reflects the intrinsic uncertainty associated with SNR estimation under noise-limited measurement conditions. In contrast, in the high-SNR regime, the remaining discrepancy between theory and experiment is limited to approximately 3 bits/s/Hz and is primarily attributed to residual misalignment-related effects in the experimental setup (see Figure S6). Variations in the incident source angles and the relative positioning of the multiplexing metasurface reduce the effective mode overlap and received signal power. Consequently, a slightly reduced achievable rate is observed experimentally at high SNR.

## Conclusions

In this work, we designed and fabricated an OAM metasurface using 72 discrete phase steps of 5°, employing Fabry–Perot-like cavity meta-atoms that provide higher transmission efficiency compared to conventional metasurfaces. For proof-of-concept demonstration, we implemented an OAM-MDM communication system incorporating both OAM multiplexing and demultiplexing metasurfaces. A channel model was developed based on ASM-based numerical simulations, and the achievable data rate was evaluated by comparing simulation and experimental results while varying the input power. In the current measurement setup, a single E-band source is divided into two beams using a power divider to validate the angle-based communication concept. Moving forward, we plan to extend this work by introducing two independently modulated E-band sources. In addition, dedicated detectors will be placed at the center of each OAM beam to enable the extraction of communication parameters for advanced modulation formats such as phase shift keying and quadrature phase shift keying. These developments aim to further advance metasurface-based OAM-MDM systems toward practical, high-capacity free-space communication.

## Methods

### Meta-atom simulation

The electromagnetic response of the meta-atom was simulated using the CST Studio Suite 2022. Simulations were performed in the frequency domain over the range of 75 GHz to 90 GHz. Both TE₀₀ and TM₀₀ modes were used as the excitation sources through waveguide ports. A tetrahedral mesh was employed, with a mesh density of 20 cells per wavelength within the meta-atom structure and 10 cells per maximum meta-atom box edge. For the background region, a coarser mesh of 4 cells per wavelength and 1 cell per maximum meta-atom box edge was applied. The simulation produced the amplitude and phase of the transmitted cross-polarized electric field over the designated frequency range.

### Fabrication of metasurface

Both the multiplexing and demultiplexing metasurfaces were fabricated using a standard printed circuit board (PCB) process. Two RF-35A2 substrates were bonded together using a bonding film (DS-7409DV(n)), resulting in a final metasurface size of 264 × 264 mm^2^. To facilitate sample handling and mounting, a 12 mm-wide border was added around the metasurface, and twelve holes with a diameter of 6 mm were drilled along the edges. To prevent oxidation on the metasurface array region, a 50 nm-thick electroless nickel immersion gold (ENIG) layer was applied. The border area was additionally coated with a photoimageable solder resist.

### Experimental demonstration

The experimental characterization was divided into two parts: OAM multiplexing and OAM demultiplexing. We first characterized the OAM generation performance of the multiplexing metasurface by excluding the demultiplexing metasurface from the setup shown in Fig. 3a. In the transmitter part, an E-band frequency extender (OML, Inc., WR10) was connected to a vector network analyzer (Keysight PNA-X N5247A) to generate a vertically polarized electromagnetic wave in the frequency range of 75–110 GHz. The E-band signal was divided using a power divider (Comotech Corp., PD1220A-H), and two conical horn antennas were connected to the output ports of the divider. Each horn antenna generated a Gaussian beam with a beam radius of approximately 8 mm and was positioned 50 mm away from a parabolic mirror. After reflection by the parabolic mirrors, the two Gaussian beams were incident onto the multiplexing metasurface at angles of ± 19.5° with respect to the z-axis, as illustrated in Fig. 3(a). Upon transmission through the multiplexing metasurface, the generated OAM beams were measured at a distance of 400 mm from the metasurface. In the receiver part, another E-band frequency extender (OML, Inc., WR10) equipped with a waveguide probe was connected to the vector network analyzer. The complex electric-field (E-field) amplitude and phase distributions of the generated OAM beams were scanned over a 200 × 200 mm^2^ area with a spatial resolution of 1 mm along the x- and y-directions in the transverse plane.

For the OAM demultiplexing measurements, the demultiplexing metasurface was inserted into the setup following the multiplexing measurement, as illustrated in Fig. 3(a). The demultiplexing metasurface was positioned 407 mm away from the multiplexing metasurface. After transmission through the demultiplexing metasurface, the deflected OAM and Gaussian beams, propagating at angles of ± 19.5° along the y-axis, were measured at a distance of 365 mm using a receiving waveguide probe connected to the E-band frequency extender. The complex electric-field amplitude and phase distributions were scanned over an area of 50 × 320 mm^2^ with a spatial resolution of 1 mm along the x- and y-directions in the transverse plane.(see the Figure S7) To suppress unwanted interference caused by scattering from the conical horn antennas and the receiving waveguide probe, anechoic absorbers were attached to the relevant components throughout all experimental measurements.

## Supplementary Information

Below is the link to the electronic supplementary material.

Supplementary Material 1

## Data Availability

All data supporting the findings of this study are available within the paper and its Supplementary Information.
