# Supplementary material for: Achievable rate analysis of orbital angular momentum multiplexing and demultiplexing using E-band metasurfaces
Source: Sci Rep. 2026 Feb 19;16:9826. doi: 10.1038/s41598-026-40149-7 (PMC13018181; doi:10.1038/s41598-026-40149-7)
Supplement: Supplementary file 1 — Supplementary Material 1 [file 41598_2026_40149_MOESM1_ESM.docx]

**Supporting Information**

Achievable rate analysis of orbital angular momentum multiplexing and demultiplexing using E-band metasurfaces

**Hyeongju Chung^1*^, Beomjoon Kim^1^, Young-Seok Lee^2^, Hongeun Choi^1^, Bang Chul Jung^3^, Eunmi Choi^1^ and Jongwon Lee^1*^**

1 Department of Electrical Engineering, Ulsan National Institute of Science and Technology (UNIST), Ulsan 44919, Republic of Korea

2 Department of Artificial Intelligence Convergence Network, Ajou University, Suwon, 16499, Republic of Korea

3 Department of Electrical and Computer Engineering, Ajou University, Suwon 16499, Republic of Korea

*E-mail: [jhj2315@unist.ac.kr](mailto:jhj2315@unist.ac.kr)

*E-mail: [jongwonlee@unist.ac.kr](mailto:jongwonlee@unist.ac.kr)

**Note 1: Angular Spectrum Method**

The Angular Spectrum Method^1-3^ (ASM) decomposes a complex wave field into an infinite set of plane waves with arbitrary propagation directions. Each plane wave is then propagated over a certain distance, and the complex wave field at the target plane is reconstructed by superimposing all the propagated components. This method accurately accounts for both propagating and evanescent components, enabling precise near-field calculations.

To simulate the propagation of a paraxial Gaussian beam in an off-axis configuration as illustrated in the measurement setup of Figure 3. We used the following formulation. The incident Gaussian beam at z=0 for each beam is defined as:

$U\left( x,y;z=0 \right)=\sum_{k=1}^{2} E_{0}exp\left( -\frac{\left( x-x_{k} \right)^{2}+y^{2}}{\omega_{0}^{2}} \right) exp\left( \left( -1 \right)^{k+1} \frac{2\pi}{\Gamma}x \right)$ (S1)

Here, E_0_​ is the amplitude of the Gaussian beam, and x, y are the spatial coordinates. ω_0_ ​ represents the beam waist radius of the Gaussian beam, which is 8 mm at 83 GHz. x_k_​ indicates the lateral position of each k^th^beam along the x-axis, and Γ​ denotes the supercell period(8×1.35mm) introduced for beam steering based on generalized Snell’s law^4^.

The propagated electric field after a propagation distance Δz is calculated using the angular spectrum method as:

$U\left( x,y;z+\Delta z \right)=\mathcal{F}^{-1}\left\{ H\left( f \right)\mathcal{\times F}\left\{ U\left( x,y;z \right) \right\} \right\}$ (S2)

$\mathcal{F}$ denote the Fourier transform operator and H(*f*) is the transfer function of free-space propagation, given by:

$H\left( f \right)=e^{ik_{0}\Delta z}e^{-i\pi\lambda\Delta z(f_{x}^{2}+f_{y}^{2})}$ (S3)

Here, k_0_​ is the free-space wavevector, and f_x ,_f_y_​ are the spatial frequencies along the x and y directions. This formulation enables accurate computation of the free-space propagated field at the target plane.

The phase functions of the OAM multiplexing and demultiplexing are given by the transmission phases in Equation (2) and (3) of the main text and can be expressed as:

$\phi_{mux}= \sum_{m=1}^{2} (l_{m}{tan}^{-1} \left( \frac{y}{x} \right)+k_{xm}x)$ (S4)

$\phi_{demux}=\sum_{n=1}^{2} {(l}_{n}{tan}^{-1} \left( \frac{y}{x} \right)+k_{yn}x+\varphi_{n})$ (S5)

After propagation through the multiplexing and demultiplexing metasurfaces located at positions z_1_=670mm and z_2_=1070mm with a thickness of t=1.164mm, the resulting field are described by:

$U\left( x,y;z+\Delta z \right)=\mathcal{F}^{-1}\left\{ H\left( f \right)\mathcal{\times F}\left\{ U\left( x,y;z \right) \right\} \right\}\times e^{j\phi_{mux}} z_{1}\leq z<z_{1}+t$ (S6)

$U\left( x,y;z+\Delta z \right)=\mathcal{F}^{-1}\{H\left( f \right)\mathcal{\times F\{}U\left( x,y;z \right)\}\}\times e^{j\phi_{demux}} z_{2}\leq z<z_{2}+t$ (S7)

This ASM-based simulation framework enables comprehensive modeling of both the generation of structured light beams carrying orbital angular momentum via multiplexing metasurface and their subsequent decomposition back into fundamental modes through demultiplexing metasurface. This approach ensures accurate phase evolution tracking across near- and far-field region.

**Note 2: OAM mode purity calculation method**

OAM mode purity of the generated beams from the multiplexing metasurface was analyzed. The circular regions indicated by the white dotted curves in Figure 4 correspond to the main intensity distributions of the generated OAM modes. The OAM mode spectrum was obtained using Fourier transform relation between angular position and OAM spectrum, which can be expressed as:

$A_{l}=\frac{1}{2\pi}\int_{-\pi}^{\pi} \Psi\left( \varphi\right)\cdot e^{-il\varphi}d\varphi$ (S8)

$\psi\left( \varphi\right)=\sum_{l} A_{l}\cdot e^{-il\varphi}$ (S9)

where φ denotes the azimuthal angle in the transverse plane of measured and simulated complex field distribution, and ψ(φ) represents the complex angular field extracted along white dotted curves in Figure 4. Here A_l_ is the OAM mode amplitude corresponding to the topological charge *l*, obtained by projecting the angular field distribution onto the basis of OAM eigenmodes. The purity of the OAM mode with topological charge *l*=*l*_0_ (l_a_=-∞<l_0_< l_b_=+∞) can be calculated as:

$OAM purity=\frac{A_{l_{0}}}{\sum_{l_{a}=-\infty}^{l_{b}=+\infty} A_{l}}$ (S10)

Where *l*_a_ and *l*_b_ denote the lower and upper bounds of the OAM mode analysis range. In this analysis, OAM mode index range was fixed to l_a_=-5 and l­_b_=+5, because power of modes above |l|=5 are very weak. As shown in Figure S4(a) and (b), the OAM mode purity of beams generated using a single source exhibits excellent agreement between the simulation and experimental result. In contrast, when two sources were simultaneously incident on the multiplexing metasurface, a slight discrepancy between simulation and experiment was observed, as shown in Figure S4(c). Specifically, due to residual misalignment between the two incident beams, the experimentally measured power ratio of the OAM l = 1 mode was approximately 15% higher than that of the OAM l = 2 mode, compared to the simulated result.

**Intensity of Gaussian beam and transmitted OAM mode from multiplexing metasurface at 83GHz**


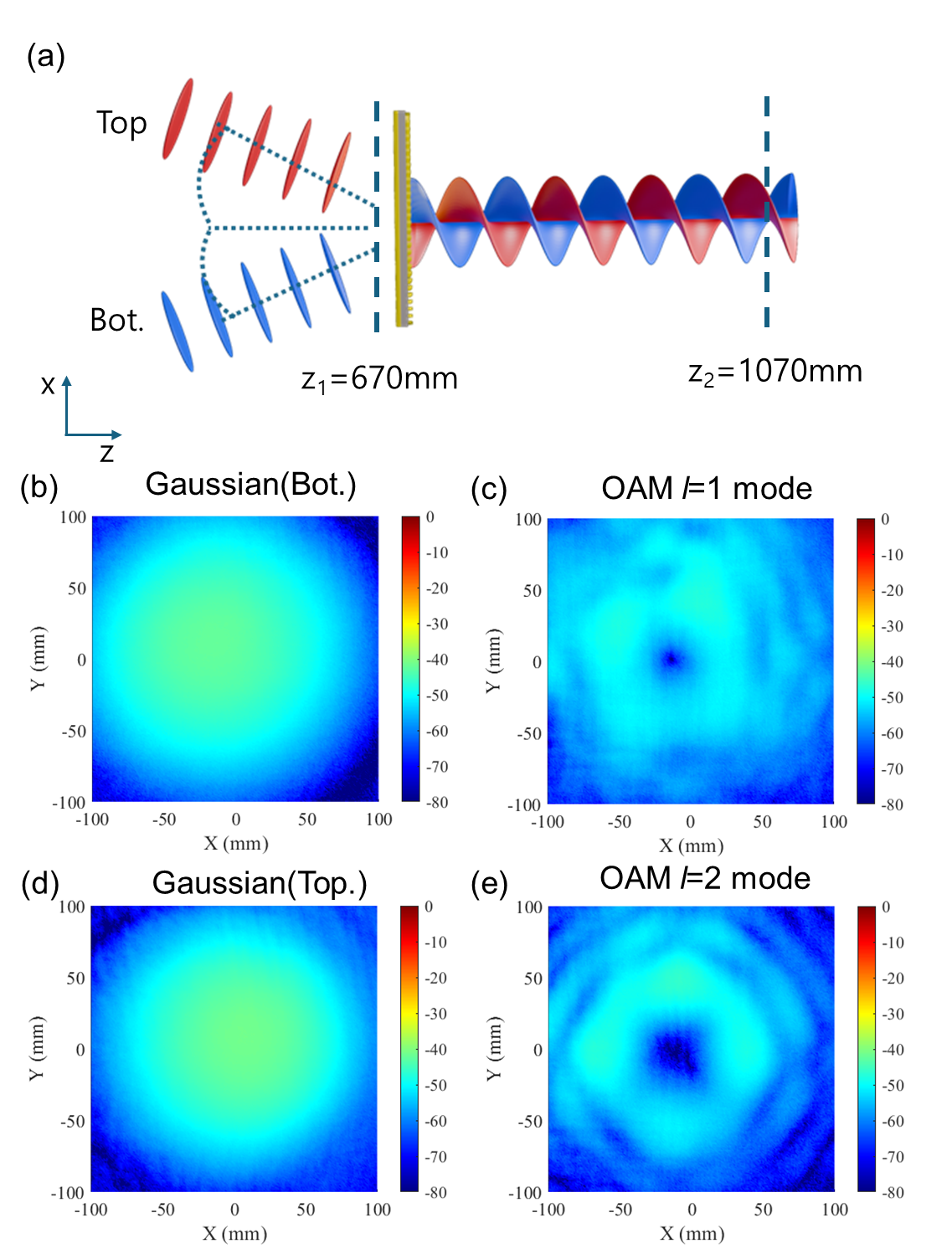


**Figure S1**. (a) Schematic of measurement set-up (b) Measured intensity of Gaussian beam when only Source 1 is incident to the multiplexing metasurface at z_1_=670mm as shown in Figure S1(a) measurement set-up. (c) Measured intensity of OAM *l*=1 mode after transmssion of multiplexing metasurface at z_2_=1070mm as shown in Figure S1(a) measurement set-up. (d) Measured intensity of Gaussian beam when only Source 2 is incident to the multiplexing metasurface at z_1_=670mm as shown in Figure S1(a) measurement set-up. (e) Measured intensity of OAM *l*=2 mode after transmssion of multiplexing metasurface at z_2_=1070mm as shown in Figure S1(a) measurement set-up.

**Transmitted OAM efficiency result from 75GHz to 90GHz**


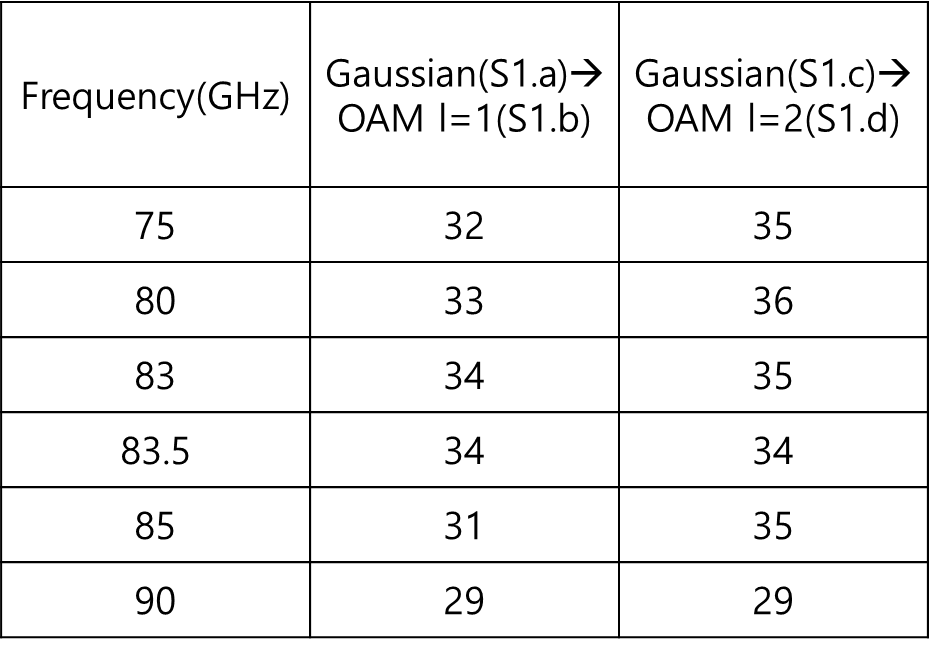


**Table S1**. Transmitted OAM efficiency^5^ is defined as $\eta_{t}=\frac{\iint\left| E_{t} \right|^{2}dxdy}{\iint\left| E_{i} \right|^{2}dxdy}\times100$, where *E_i_* and *E_t_* represent the measured electric field at the incident side (Gaussian beam) and transmission side(OAM beam), respectively. x and y is the spatial coordinate of measured section as shown in Figure S1.

**OAM mode purity analysis of generated beams from multiplexing metasurface**

**
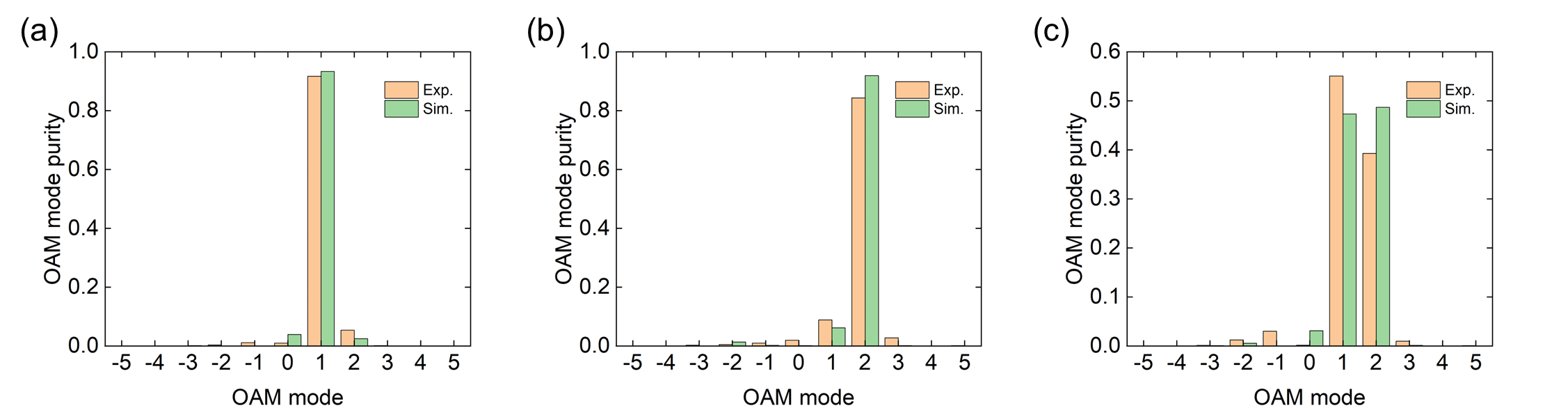
**

**Figure S2.** Experiment and simulation result of OAM mode purity analysis for OAM *l*=1(a), OAM *l*=2(b) and OAM *l*=1&2(c).

**Note 3. Sensitivity analysis for incidence angle of source and multiplexing metasurface position**

To quantitatively investigate the sensitivity of the proposed OAM multiplexing scheme to alignment errors, we performed a systematic numerical simulation–based sensitivity analysis with respect to the incidence angle of the input sources and the lateral position of the multiplexing metasurface. The analysis focuses on the two generated OAM channels, $l=1$and $l=2$, and evaluates both the OAM mode purity and the associated inter-modal crosstalk under controlled parametric variations.

The total OAM crosstalk is defined based on the relative power leakage into undesired OAM modes as

$$\begin{matrix} & \mathrm{XT}_{\mathrm{tot}}\text{ }(\mathrm{dB})=10\log_{10}\left( \frac{1-\left( P_{l=1} + P_{l=2} \right)}{P_{l=1}+P_{l=2}} \right), & & \text{(S11)} \end{matrix}$$

where $P_{l=1}$and $P_{l=2}$denote the normalized modal power of the target OAM modes extracted from the simulated OAM purity analysis.

First, the incidence angle of input source 2 was fixed at ${19.5}^{\circ}$, while the incidence angle of input source 1 was varied from ${15.5}^{\circ}$to ${23.5}^{\circ}$in steps of $2^{\circ}$within the numerical model. The corresponding simulated OAM purity distributions and crosstalk values are shown in Figure S3(a) and Figure S3(b), respectively.

At the nominal incidence angle of ${19.5}^{\circ}$, the simulated OAM beams exhibit balanced modal purity for $l=1$and $l=2$, resulting in the minimum crosstalk level. As the incidence angle deviates from this optimal condition, the relative modal balance between the two OAM channels deteriorates, accompanied by a pronounced increase in crosstalk. This trend originates from the effective lateral shift of the beam footprint on the multiplexing metasurface induced by angular deviation, which leads to non-uniform excitation of the designed phase-gradient distribution and consequently degrades the OAM separation efficiency.

Next, both input sources were fixed at incidence angles of $\pm{19.5}^{\circ}$, while the multiplexing metasurface was translated along the y-axis from −7.5 mm to +7.5 mm with a step size of 2.5 mm in the simulation. The resulting OAM purity and crosstalk characteristics are presented in Figure S3(c) and Figure S3(d).

The simulation results indicate that optimal OAM mode purity is achieved when the metasurface is centered at y-shift = 0. Any displacement along the y-axis leads to a breakdown of the intended modal balance between the generated OAM channels. Correspondingly, the simulated crosstalk increases symmetrically as the metasurface is shifted away from the center position. This symmetric dependence confirms that the observed degradation is governed primarily by geometrical misalignment effects inherent to the multiplexing configuration, rather than by asymmetric scattering or numerical artifacts.

Finally, the incidence angles of the input beams were fixed at $\pm{19.5}^{\circ}$, and the metasurface position was fixed at y-shift = 0, while the metasurface was translated along the x-axis from −7.5 mm to +7.5 mm in increments of 2.5 mm in the simulation. The corresponding OAM purity and crosstalk results are shown in Figure S3(e) and Figure S3(f). Consistent with the y-axis displacement analysis, the simulated results show that the highest OAM purity and lowest crosstalk are obtained when the metasurface is positioned at x-shift = 0. Deviations from this optimal position result in a rapid degradation of modal purity and a corresponding increase in inter-modal crosstalk. The symmetric behavior with respect to the x-axis further highlights the critical role of precise spatial alignment in maintaining robust OAM channel separation.

These simulation-based results indicate that deviations in incidence angle or lateral alignment inevitably modify the spatial overlap between the incident beams and the designed phase-gradient profile of the multiplexing metasurface. As a result, the intended OAM modal balance is perturbed, leading to reduced OAM mode purity and increased inter-modal crosstalk.

**OAM purity and crosstalk analysis for incidence angle of source and position of multiplexing metasurface**


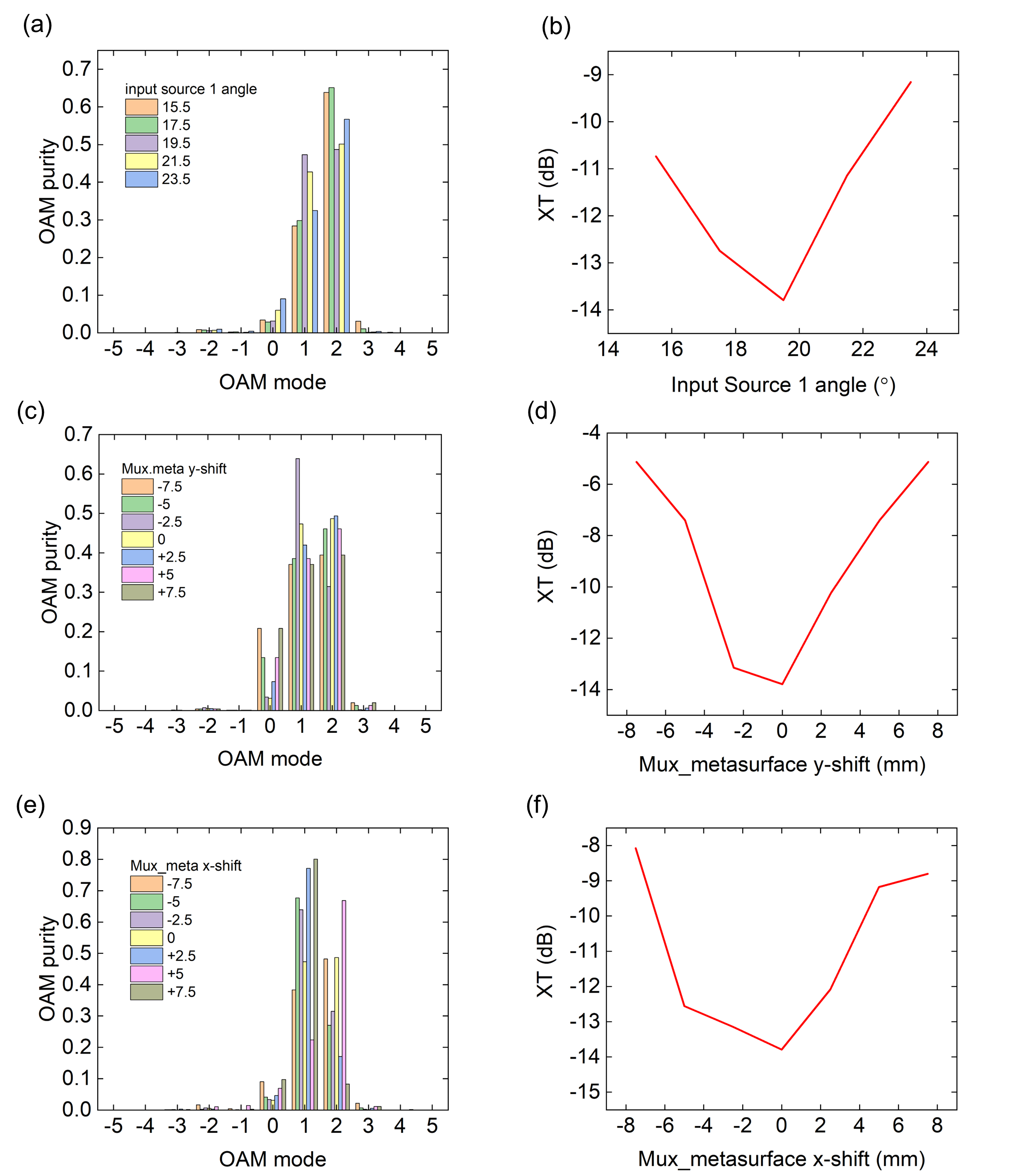


**Figure S3.** Result of (a) OAM purity and (b) crosstalk according to the input incidence angle of source 1. Result of (c) OAM purity and (d) crosstalk according to the multiplexing metasuface y-directional position. Result of (c) OAM purity and (d) crosstalk according to the multiplexing metasuface x-directional position.

**Amplitude and phase of simulation from demultiplexing metasurface at 83GHz**


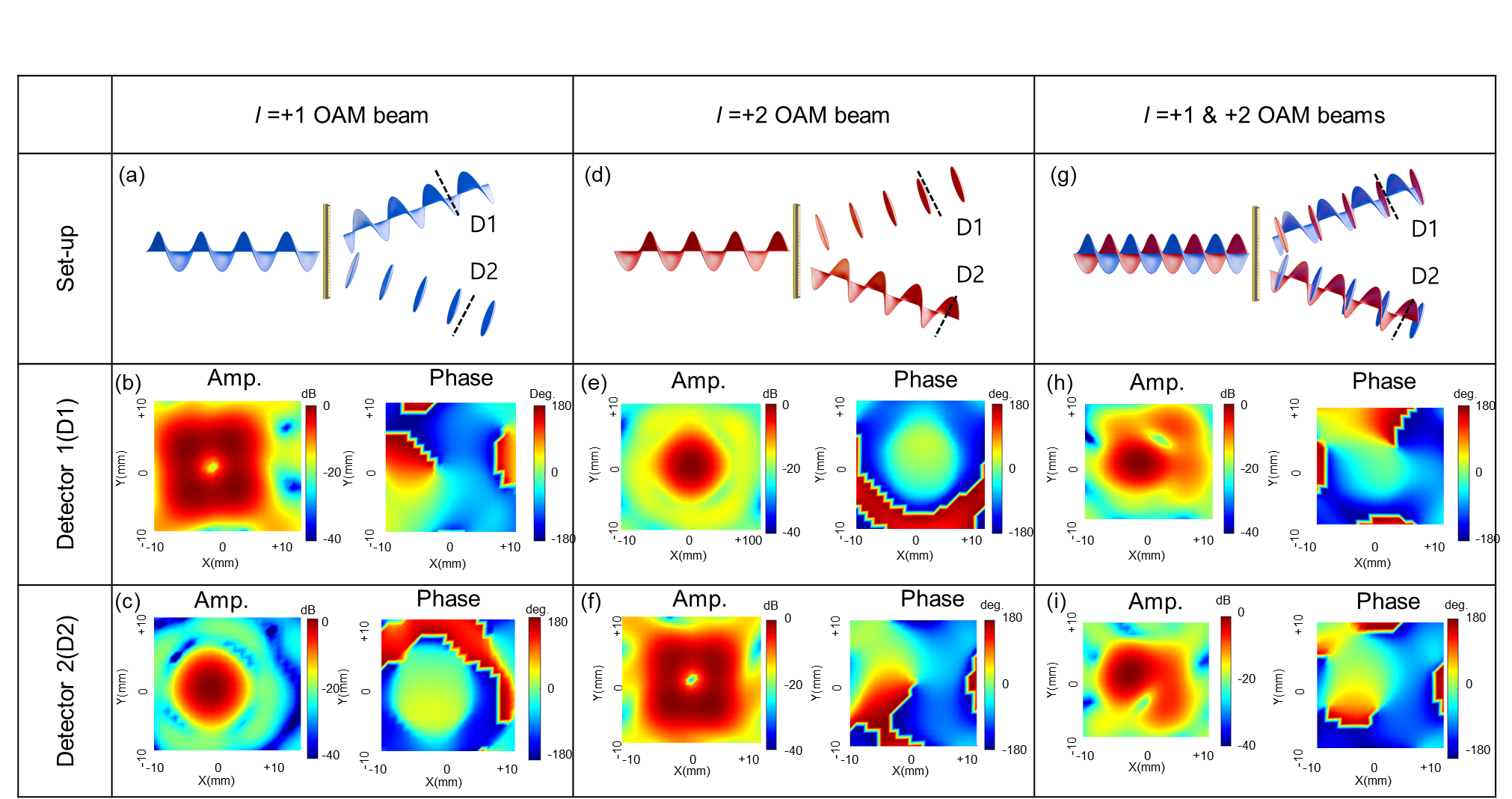


**Figure S4.** (a) simulation set-up for demultiplexing of OAM l=+1. (b) amplitude and phase of OAM l=-1 beam at detector 1. (c) amplitude and phase of Gaussian beam at detector 2. (d) simulation set-up for demultiplexing of OAM l=+2. (e) amplitude and phase of Gaussian beam at detector 1. (f) amplitude and phase of OAM l=+1 beam at detector 2. (g) simulation set-up for demultiplexing of combined OAM l=+1&+2. (h) amplitude and phase of combined OAM l=-1&Gaussian beam at detector 1. (i) amplitude and phase of Gaussian beam&OAM l=+1 beam at detector 2.

**Mode purity analysis from simulation of demultiplexing metasurface in Figure S4.**


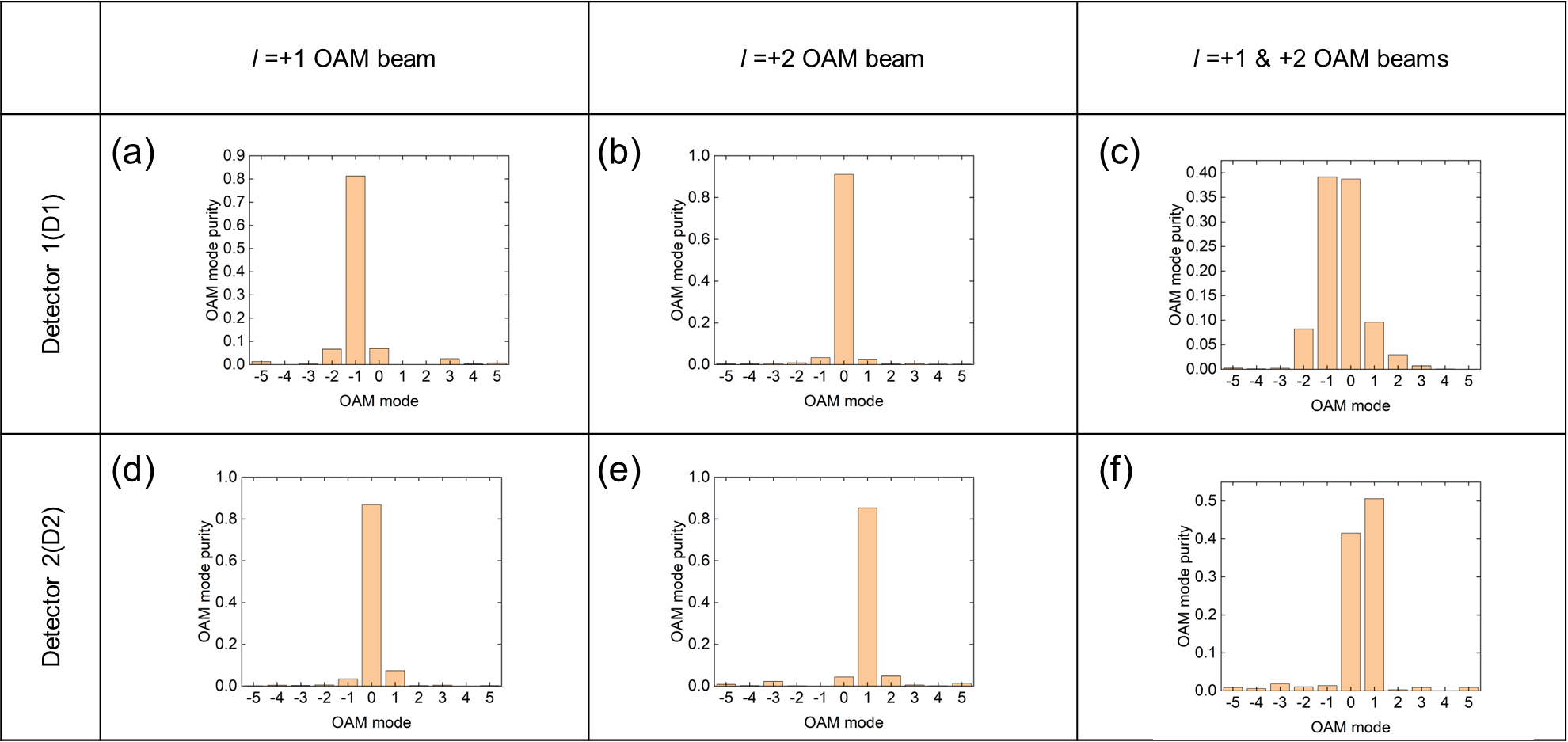


**Figure S5.** (a, d) Mode purity result from Figure S2(b,c). (b,e) Mode purity result from Figure S2(e,f). Mode purity result from Figure S2(h,i).

**Achievable rate analysis according to the input incidence angle and multiplexing metasurface position.**

**
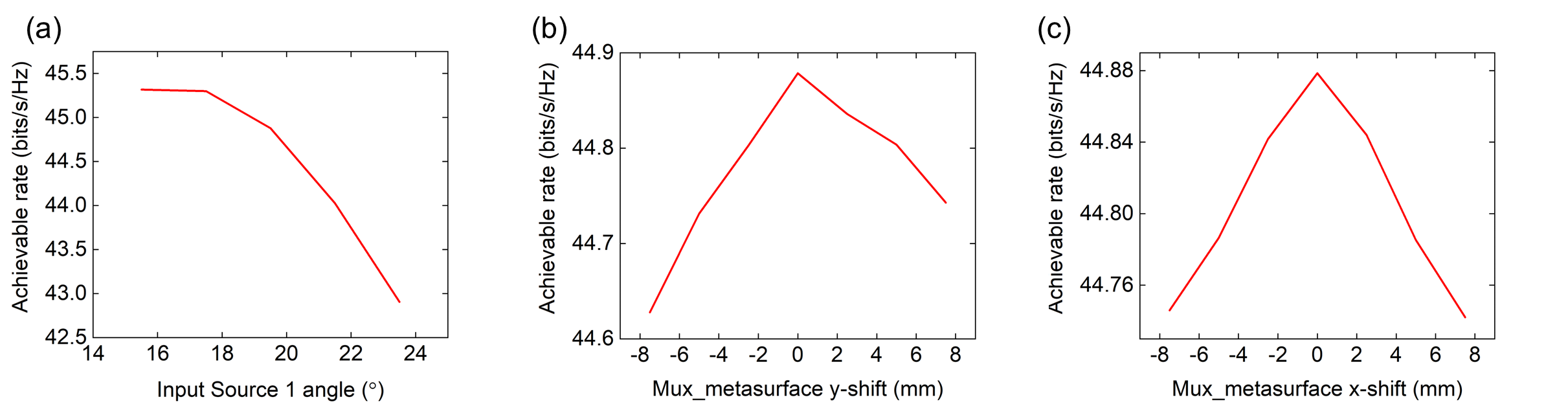
**

**Figure S6.** Achievable rate evaluated after transmission through the demultiplexing metasurface under variations of the input incidence angle and the lateral position of the multiplexing metasurface. (a) Achievable rate as a function of the incidence angle of input source 1, while the incidence angle of input source 2 is fixed at 19.5°. (b) Achievable rate as a function of the lateral displacement of the multiplexing metasurface along the y-axis, with both input sources fixed at incidence angles of ±19.5°. (c) Achievable rate as a function of the lateral displacement of the multiplexing metasurface along the x-axis, with the same incidence-angle configuration.

**Measured amplitude and phase of simulation from demultiplexing metasurface at 83GHz**


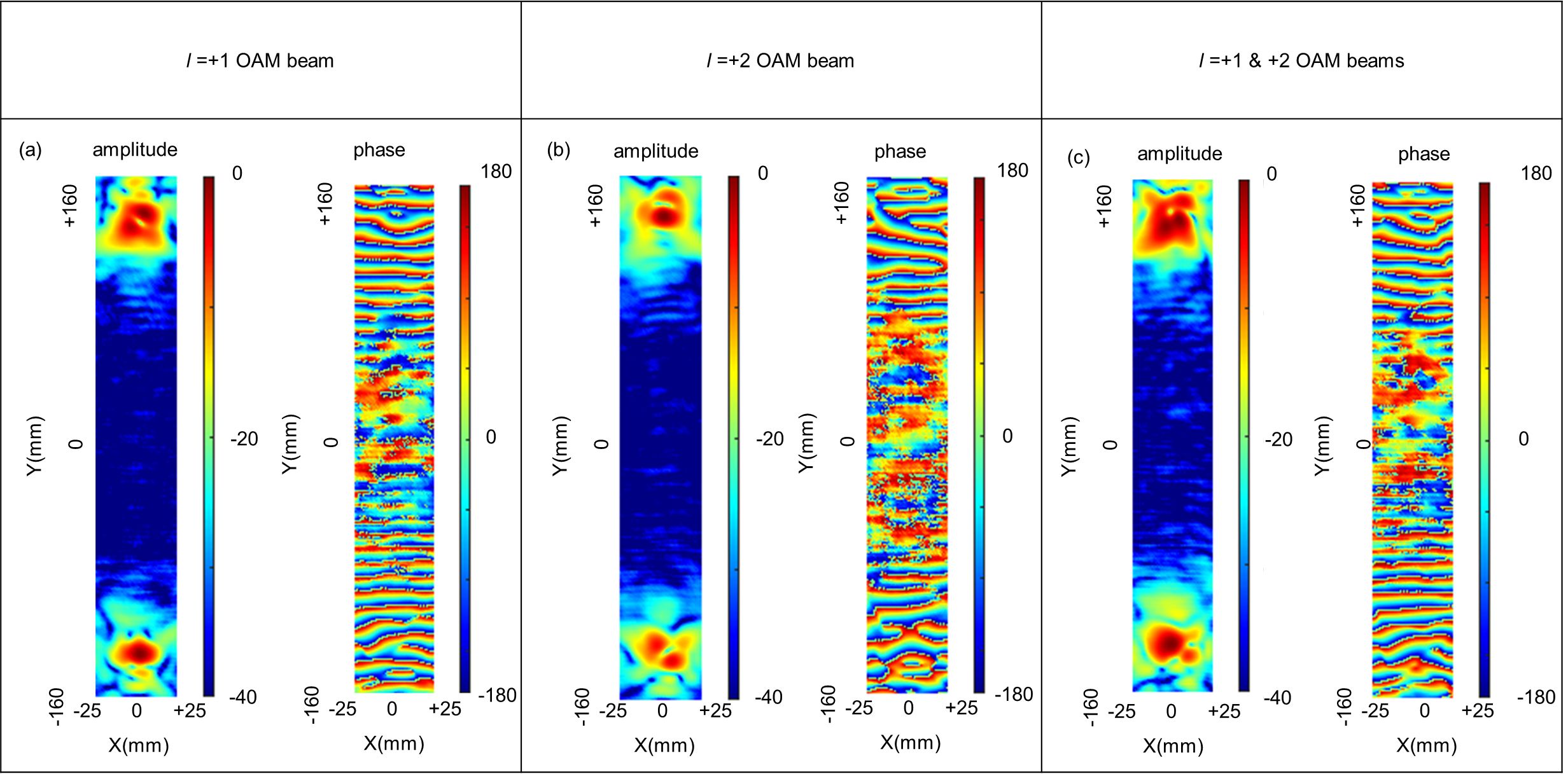


**Figure S7.** (a) Measured amplitude and phase for demultiplexing of OAM +1 and the scanned area is 50 × 320 mm². (b) Measured amplitude and phase for demultiplexing of OAM +2 and the scanned area is 50 × 320 mm². (c) Measured amplitude and phase for demultiplexing of combined OAM l=+1&OAM +2 and the scanned area is 50 × 320 mm².

References

1 Matsushima, K. Shifted angular spectrum method for off-axis numerical propagation. *Optics Express* **18**, 18453-18463 (2010).

2 Matsushima, K. & Shimobaba, T. Band-limited angular spectrum method for numerical simulation of free-space propagation in far and near fields. *Optics express* **17**, 19662-19673 (2009).

3 Sawant, A., Lee, I. & Choi, E. Amplitude non-uniformity of millimeter-wave vortex beams generated by transmissive structures. *IEEE Transactions on Antennas and Propagation* **70**, 4623-4631 (2022).

4 Yu, N. *et al.* Light propagation with phase discontinuities: generalized laws of reflection and refraction. *science* **334**, 333-337 (2011).

5 Akram, M. R. *et al.* Photon spin Hall effect-based ultra-thin transmissive metasurface for efficient generation of OAM waves. *IEEE Transactions on Antennas and Propagation* **67**, 4650-4658 (2019).
